# Constructing more comprehensive pollination networks: integrating diurnal and nocturnal pollen data with visitation in a subalpine wetland community

**DOI:** 10.3389/fpls.2024.1464970

**Published:** 2024-10-08

**Authors:** Yue Teng, Jana C. Vamosi, Xiao-Fan Wang, Yan-Bing Gong

**Affiliations:** ^1^ State Key Laboratory of Hybrid Rice, Key Laboratory of Biodiversity and Environment on the Qinghai-Tibet Plateau, Ministry of Education, College of Life Sciences, Wuhan University, Wuhan, China; ^2^ Department of Biological Sciences, University of Calgary, Calgary, AB, Canada

**Keywords:** light trap, modularity, network structure, plant-pollinator interactions, pollen analysis, sample effort

## Abstract

**Introduction:**

Sampling for describing plant–pollinator interaction networks has been performed using techniques that either focus on the plants (with flower-visit data) or the animals (with analyzing pollen on the body surface of flower visitors). The differences in the structure of the networks obtained using these methods likely influences our understanding of the contribution of nocturnal pollinators, yet this key finding has yet to be the focus of study.

**Methods:**

In this study, we conducted an intensive diurnal field survey in the subalpine meadows of the Dajiuhu Wetland and supplemented the data with an analysis of diurnal and nocturnal pollen data to examine the changes in pollination networks.

**Results:**

We observed 41 plant and 154 pollinator species, corresponding to 665 specific interactions. Visitation and pollen analyses showed significant differences in the composition and interaction between network plants and pollinators, resulting in important structural changes in the network. Given that the diurnal pollen data showed new links that were preferentially attached to highly connected nodes, the level of asymmetric specialization did not decrease; however, nestedness increased 1.3-fold, and mean pollinator connectivity from 3.1 to 5.1. As the behaviors of nocturnal pollinators tended to be more specialized, the inclusion of nocturnal pollen data led to an increase in the number of extreme-specialist pollinator species. Consequently, nestedness decreased 0.8-fold, but mean plant connectivity went from 14.2 to 16.2.

**Discussion:**

These findings suggest that the structure of pollination networks is influenced by the sampling methods and the level of detail of the investigation. Our study has strong implications for the development of monitoring schemes for plant–pollinator interactions. Due to the practical difficulties of nocturnal field visitation, when conducting research, combining diurnal field visitation with both diurnal and nocturnal pollen analyses is the most convenient and realistic method to capture the full complexity of these networks.

## 1 Introduction

Pollinators help stationary plants deliver pollen to exchange gametes with other plants or with themselves (Loy and Brosi, 2022). Flowering plants offer food resources to pollinators in exchange for pollination services (Ollerton et al., 2011). There is global concern about the recently observed declines in the diversity and distribution of pollinators and the consequences these declines will have for pollination services (Potts et al., 2010; Janousek et al., 2023). Given that the reproductive success of up to 94% of flowering plants can be affected (Ollerton et al., 2011) by pollinator loss, it is clear that pollinators play a critical role in maintaining plant biodiversity, ecosystem stability, and resilience (Bronstein et al., 2006; Ashworth et al., 2009; Memmott, 2009; Vázquez et al., 2009; Potts et al., 2010). This has created an urgent need to monitor plant and pollinator diversity and characterize the interactions between them. Interaction network approaches at the community level can be used to rapidly examine the interactions between plants and their potential pollinators, and provide insight into whether there are general rules governing which pollinators contribute most to these ecological services (Baldock et al., 2011; Chamberlain et al., 2014). However, if the pollination network obtained is not sufficiently detailed, the rules obtained are biased. Therefore, identifying the best approaches for pollinator monitoring is an important goal in ecology.

Although nocturnal animals have been overlooked in previous studies, they play a vital role in pollination and sexual reproduction in plants (Teng et al., 2024). Among them, moths are nocturnal pollinators that can carry pollen over greater distances than diurnal insect pollinators (Devoto et al., 2011; Macgregor et al., 2017). Moths contribute significantly to pollination by facilitating higher quality plant production during visits, surpassing the effectiveness of other pollinators (Devoto et al., 2011; Fijen et al., 2023). They play a crucial role in the pollination of non-crop plants and are essential for the preservation of biodiversity within ecosystems (Hahn and Brühl, 2016). Although some researchers have recently begun to focus on nocturnal pollination (Knop et al., 2017, Knop et al., 2018; Walton et al., 2020; Giavi et al., 2021; García et al., 2024), it has often been overlooked, partially because of the intrinsic difficulty of field experimentation at night. Nocturnal field visitation surveys are undoubtedly more difficult, especially at the community level, and only a few surveys have gathered direct observations (Knop et al., 2017, Knop et al., 2018). Alternatively, light traps are widely used to attract nocturnal insects because they allow large numbers of specimens to be caught with minimal effort (Atwater, 2013; Banza et al., 2015; Hahn and Brühl, 2016). However, the relationship between insects and plants cannot be determined by using light traps alone. This problem can be solved by nocturnal pollen analysis (identification of plant species visited by nocturnal insects using pollen from the bodies of pollinators) after nocturnal insects are captured using light traps (Devoto et al., 2011; Souza et al., 2022). García et al. (2024) constructed the nocturnal pollen network by the methods above and suggested that ignoring the nocturnal component of plant−pollinator networks may cause changes in network properties different from those expected from random undersampling of diurnal pollinators (**Table 1**).

**Table 1:** Comparison of characteristics of visitation (V) and insect pollen load (P) networks in seven related studies.

Studies	García et al., 2024	Cirtwill et al., 2024	Tourbez et al., 2023	de Manincor et al., 2020	Zhao et al., 2019	Alarcón, 2010	Bosch et al., 2009	This study	
Analysis	V_d_ vs V_d_P_n_	V vs P	V vs P	V vs P	V vs P	V vs P	V vs P	V_d_ vs P_d_	V_d_P_d_ vs V_d_P_d_ P_n_
Plant species	↑	↑	↑	↑	n.a.	↓	↓	↑	―
Pollinator species	↑	↓	―	―	↓	↓	↓	↓	↑
Interactions recorded	↑	↑	↑	↑	↓	↓	↑	↑	↑
Connectance	↓	↑	↑	↑	n.a.	↑	↑	↑	↓
Mean plant connectivity	↓	↑	↑	n.a.	n.a.	↓	↑	↓	↑
Mean animal connectivity	↓	↑	↑	n.a.	n.a.	↓	↑	↑	↓
% Extreme animal specialists	↑	↓	↓	↓	↑	n.a.	↓	↓	↑
NODF	↓	↑	↑	n.a.	↓	n.a.	↑	↑	↓
Degree centralization	n.a.	n.a.	n.a.	n.a.	n.a.	n.a.	↑	↓	↓
Modularity (*M*)	↑	n.a	↓	n.a.	↑	n.a.	―	↓	↑
Number of significant modules	n.a.	n.a.	n.a.	n.a.	n.a.	n.a.	↑	↓	↑

‘n.a.’ data not included in the study. ‘↑’, ‘↓’, and ‘―’ represent increased, decreased, and not changed effects, respectively. Since the nocturnal pollen network was involved in this study, the networks before and after adding the nocturnal pollen data were also compared.

Sampling bias often accounts for some of the data gaps found and can, therefore, influence pollinator network structure (Petanidou et al., 2008). Pollinator monitoring can be accomplished through a variety of methods, most of which are based on direct observations, including transects and observation of contact between visitors and flowers in the field. Plant and pollinator abundances are usually thought to be important, although some rare species can remain undetected (Gómez et al., 2007). More intense sampling can reduce the probability of missing some interactions but can increase the time and personnel costs of the experiment (Olesen et al., 2011). Using visual surveys combined with pollen from the bodies of pollinators (pollen analysis) is an alternative method for comparing the two sampling methods (Bosch et al., 2009; Alarcón, 2010; Dorado et al., 2011; Olesen et al., 2011; Popic et al., 2013; Zhao et al., 2019; de Manincor et al., 2020; Tourbez et al., 2023; Cirtwill et al., 2024). These studies have highlighted the effects of sampling on network structure (summarized in **Table 1**). Most studies have suggested that by adding the pollen data, the connectance, nestedness and connectivity of plants and pollinators increased, and the number of extreme specialists decreased (Bosch et al., 2009; de Manincor et al., 2020; Tourbez et al., 2023; Cirtwill et al., 2024). However, some studies have suggested that when ‘cheater’ pollinators—those recorded visiting certain plants but not carrying their pollen—are excluded from the pollen data, the connectivity of plants and pollinators, as well as the nestedness, decreases (Alarcón, 2010; Zhao et al., 2019).

Interactions between different plants and their pollinators can reveal structural features of interaction networks, such as nestedness, specialization, modularity, and asymmetric dependence (Vázquez et al., 2009). Numerous studies have shown that plant-pollinator networks exhibit nested structures, whereby specialists (species that interact with one or a few other species) tend to interact more frequently with certain species that are subsets of more generalized species (Bascompte et al., 2003; Olesen et al., 2008; Petanidou et al., 2008). If the degree of specialization within a community follows a truncated power law distribution, this indicates that most species are specialized, while a few species have interaction frequencies significantly above the average (Jordano et al., 2002; Vázquez and Aizen, 2004; Potts et al., 2006). Different network characteristics indicate varying ecological states. For instance, higher connectance and nestedness within a network enhance the stability of interaction networks (Thébault and Fontaine, 2010). Nested structures can buffer the temporal fluctuations in the abundance of specialized pollinators or reduce the risk of secondary extinctions caused by the loss of specialized pollinators, as a plant species can be pollinated by other more generalized species (Tylianakis et al., 2010). Increased network connectance may enhance ecosystem stability, and for networks of specific sizes, higher connectivity among involved species could provide a buffering effect against fluctuations in their interacting partners (Tylianakis et al., 2010). Modularity refers to the division of a network into modules, where interactions among species within a module are stronger than interactions with species in other modules; thus, modules can represent subnetworks within the overall network. Species within a module may exhibit convergent characteristics to some extent, and these characteristics and modules can be considered units of co-evolution among species (Olesen et al., 2008). In pollination networks, these units may correspond to species exhibiting the same pollination syndrome, specifically the composite traits of plants and their corresponding functional groups of pollinators (Fenster et al., 2004; Carstensen et al., 2014). Therefore, different network parameters can be computed to compare the structural characteristics of diverse pollination networks and to preliminarily assess network stability. Additionally, the interactions between plants and pollinators are influenced by the composition and abundance of both plants and pollinators, and changes in community structure can significantly impact plant-pollinator interactions at the community level.

In this study, we analyzed the structure of plant–pollinator interactions in a subalpine wetland community in Dajiuhu, central China, based on diurnal visitation and both diurnal and nocturnal pollen analyses, including both diurnal and nocturnal pollination networks. We aimed to test the hypotheses of complementarity and redundancy in pollination networks with the inclusion of both diurnal and nocturnal pollen data. If diurnal and nocturnal pollen data is complementary to diurnal pollination, we expect to observe new plants and pollinators involved in the network, thereby increasing the overall range of the pollination network. If diurnal and nocturnal pollen data is redundant, we expect to see the same set of pollinators interacting with different plants, or different pollinators interacting with the same plants, thus adding redundancy to the existing network. To test these hypotheses, we will compare the structure and composition of diurnal and nocturnal pollination networks. Diurnal pollen data holds the potential to complement plant–pollinator interactions not recorded by diurnal visitation data, and nocturnal pollinators are different from diurnal pollinators (Borges et al., 2016; Knop et al., 2018). We predicted that the network structure may change after adding diurnal or nocturnal pollen data to the diurnal visitation network, which will provide a more complete view of the interaction network, resulting in higher connectance and connectivity. Because diurnal pollen data may record more rare species interactions (Bosch et al., 2009; Cirtwill et al., 2024), the nestedness of the network may increase and modularity may decrease; thus, this may support the redundancy hypothesis for diurnal pollination. Simultaneously, because nocturnal pollinators are different and more specialized than diurnal ones (Fontaine et al., 2008; Devoto et al., 2011), we predicted that the addition of nocturnal pollen data decreases the nestedness and increases the modularity of the network; therefore, this aligns with the complementarity hypothesis for nocturnal pollination. Wetlands play a major role in the biosphere by providing habitats for certain plants, animals, and other life forms and may also serve as the last refuge for many rare and endangered species (LePage, 2011). Therefore, we designed this study to understand how sampling approaches affect the assessment of interaction networks that can help inform appropriate conservation strategies to preserve, maintain, and improve wetlands.

## 2 Materials and methods

### 2.1 Study site and data collection

This study was conducted in a subalpine wetland located in Dajiuhu, Shennongjia National Park (31°30′15′′N, 109°59′47′′E, 1750 m a.s.l.), in western Hubei Province, central China. The experiment was conducted during the peak flowering season from mid-July to late August 2018. The region has a climate with short, warm, and wet summers and long, cold winters because of its higher altitude. There are no plants that bloom during the winter season (November–April) (Ma et al., 2008).

**Figure 1:**
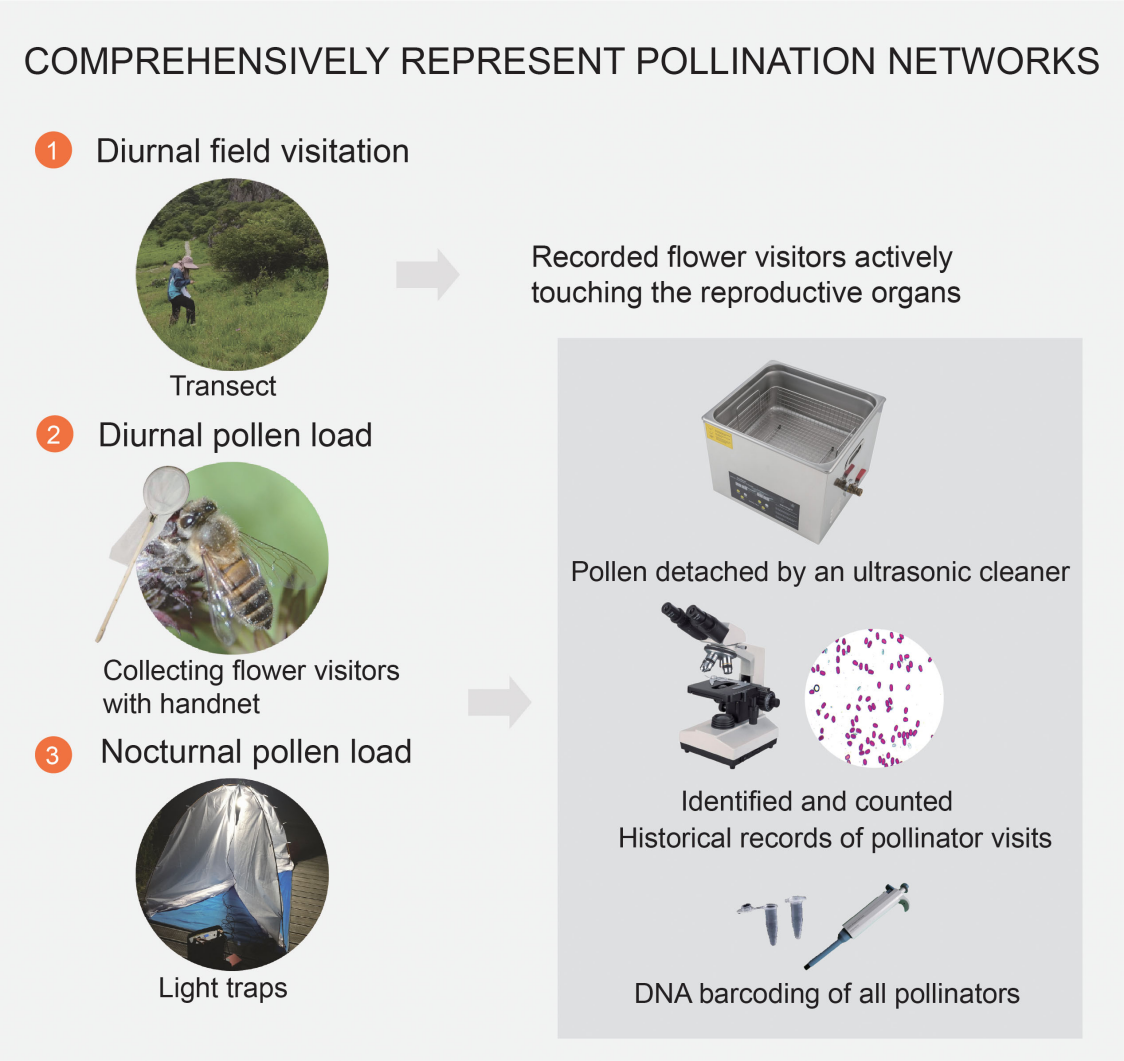
Detailed methodologies for representing pollination networks in a subalpine wetland in Dajiuhu, focusing on the specific steps of diurnal field visitation, diurnal pollen analysis, and nocturnal pollen analysis during peak flowering from July to August in 2018.

As details of the study community, including pollinator observations, nocturnal light traps and pollen analysis have been previously described by Teng et al. (2024), we present only a brief description here. All the surveys were conducted in six meadows that covered an excess of *c.* 5000 m^2^ and were *c.* 200 m apart. We observed plant-pollinator interactions at five intervals throughout the season (**Figure 1**). Each census comprised 24 observation periods, with each period lasting 15-min, resulting in a total observation time of 6 h per census (**Supplementary Materials**). The corresponding plant taxa were recorded for each visitor. To ensure accurate pollen analysis, each flower visitor was captured individually and promptly transferred to a separate vial. This precautionary measure prevented any potential pollen contamination that could occur from contact with other collected insects. Within each meadow, we randomly established nine 2 × 2 m^2^ plots to measure floral abundance and flowering phenology. Once per census, all open flowers or floral visual units were counted for all taxa present in each plot (Gong and Huang, 2009) (**Supplementary Materials**; **Supplementary Table 1**).

Nocturnal flower visitors were sampled using light traps equipped with 160 W mercury tubes powered by 12 V batteries. Selection of sampling nights was based on favorable weather conditions, with low wind speeds and no rain. Traps were strategically positioned along a path close to the meadows. They were operational from 2000 to 2400 h, encompassing a four-hour duration (Kadlec et al., 2009; Atwater, 2013). In total, six light traps were deployed, with each trap spaced at intervals of 5–10 d. To preserve diurnal and nocturnal captured insects, we placed them individually in tubes and stored them in a freezer until further processing (Bosch et al., 2009; Devoto et al., 2011).

The captured insects were washed multiple times to ensure the thorough removal of all pollen grains adhering to their bodies for pollen analysis. Pollen grains were identified with the aid of a pollen reference collection from the study area, based on the morphological characteristics of the pollen grains found in insect bodies (**Supplementary Materials**). To confirm the visitation to a particular plant species, we considered the presence of at least five pollen grains from that species in our pollen counts (Knop et al., 2017).

All flower visitors were determined to the genus or family level and, where possible, to the species level (**Supplementary Table 2**). DNA barcoding is a rapid and efficient method for species identification that analyzes DNA sequences extracted from small tissue samples of any organism (Kress and Erickson, 2012; Wilson et al., 2017). The identification process involved a combination of morphological and molecular techniques. For the molecular analysis, we used DNA barcoding methods with the CO1 gene to achieve accurate pollinator species identification whenever possible. We divided all visitors into 10 functional groups: (1) bumblebees (*Bombus* spp.); (2) honeybees (*Apis cerana*); (3) solitary bees (Halictidae, Melittidae, *Eucera* spp., Andrenidae, Braconidae, Cerceridae, Scoliidae, Ichneumonidae, Pemphredonidae, Tenthredinidae); (4) hoverflies; (5) other flies; (6) beetles; (7) stinkbugs and cicadas; (8) butterflies; (9) moths; and (10) others (ants, mosquitos, Orthoptera, Mecoptera, Trichoptera). All insect species were prepared as pinned specimens and stored at the Gong Laboratory at Wuhan University.

### 2.2 Network construction and analysis

**Figure 2:**
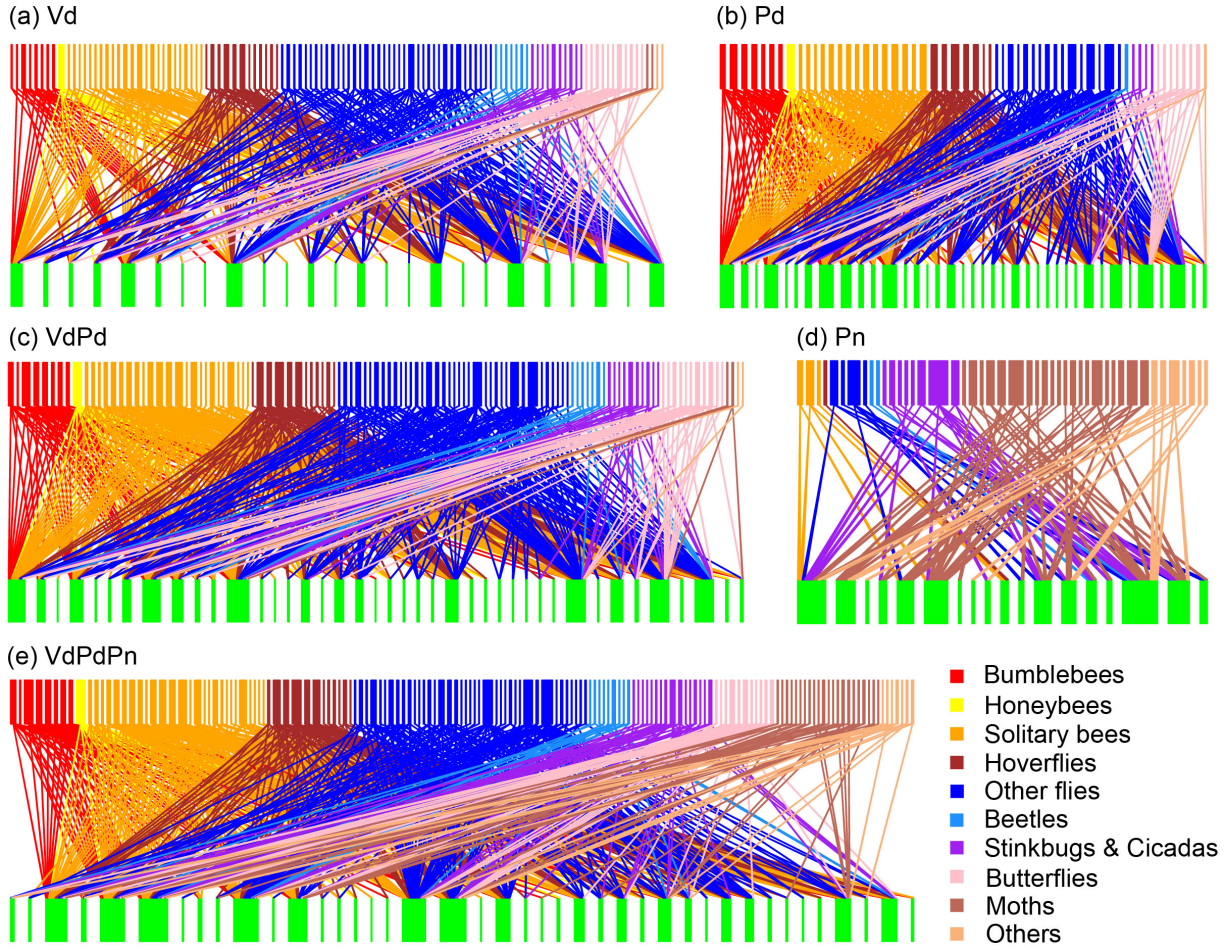
Bipartite networks illustrating the plant-pollinator interactions of **(A)** V_d_ matrix (diurnal field visitation); **(B)** P_d_ matrix (diurnal pollen analysis); **(C)** V_d_P_d_ matrix (diurnal field visitation + diurnal pollen analysis); **(D)** P_n_ matrix (nocturnal pollen analysis); and **(E)** V_d_P_d_P_n_ matrix (diurnal field visitation + diurnal pollen analysis + nocturnal pollen analysis). The rectangles represent insect species (top) and plant species (bottom), and the connecting lines represent interactions among species. The width of the boxes represent the number of types of interactions.

We built five plant–pollinator qualitative binary matrices with data from diurnal field visitation (matrix V_d_), diurnal pollen analysis (matrix P_d_), diurnal visitation and pollen analysis (matrix V_d_P_d_), nocturnal pollen analysis (matrix P_n_), and all three types of data (matrix V_d_P_d_P_n_) (**Supplementary Table 3**; **Figure 2**). The superimpose method involves sequentially adding interactions that were not recorded by the previous method to obtain a new pollination network (Bosch et al., 2009).

For each network, we calculated the following parameters using the ‘bipartite’ package (Csardi and Nepusz, 2014) in R version 4.0.2 (R Development Core Team, 2020). Connectance (network-level) is the proportion of observed links divided by the number of total possible links, whereas connectivity (species-level), represented by *s*, measures the number of interaction partners of one species (species degree), and is a measure of its generalization. Degree centralization is a measure of the level of centralization in the network, where maximum centralization (DC = 1) is reached in star networks, with a central node linked to the rest of the nodes, which are not linked between themselves (Borgatti and Everett, 1999). NODF is the tendency for specialist species to interact with generalists and the stability of plant–pollinator communities (Beckett et al., 2014). Modularity is an index of modularity that measures the extent to which species have more links within their modules than expected if linkage was random (Guimerà and Nunes Amaral, 2005). We ran the QuanBiMo algorithm following the methodology established by Schleuning et al. (2014) and the default specifications of the computeModules function in bipartite. Each species was sorted into peripheral, connector, module hubs, and network hubs (Olesen et al., 2007). To assess the significance of the two network metrics (NODF and Modularity), we compared the observed values to those generated by null models. We controlled the effect of the network size by standardizing the network links using Z scores, comparing them against 1000 random network models generated with the ‘r2dtable’ function from the Vegan package in R (Dormann and Strauss, 2014).

### 2.3 Data analysis

To differentiate the sampling approach, that is, field visitation data versus pollen data and/or the increased sampling effort, we used the R package iNEXT to generate rarefaction curves (mean ± 95% confidence intervals) of the expected accumulation of interactions with the five datasets (V_d_, P_d_, V_d_P_d_, P_n_ and V_d_P_d_P_n_).

To examine the cumulative distribution of connectivity, we used the R package to fit three different models to the distribution of connectivity in our five matrices, namely exponential, power law, and truncated power law (Jordano et al., 2002; Gillespie, 2015). Linear regression was also used to explore the relationship between *f* (interaction frequency and number of observed flower visits) and *s* (connectivity). We used ANCOVA to compare the slopes of the regression lines between *s* and *f* of the three matrices (V_d_, V_d_P_d_ and V_d_P_d_P_n_) for plants and pollinators. This was conducted to determine whether the *f*-*s* relationship changed significantly after the addition of pollen data. Linear regression was used to determine whether the increase in *s* was related to the number of specimens sampled. All censuses were log-transformed prior to analysis.

Generalized linear mixed models (GLMM) with a binomial distribution were used to compare significant differences in the percentage of within- and between-module link gain with the addition of diurnal and nocturnal pollen data among species belonging to various modules (**Supplementary Table 6**). The percentage of within- and between-module link gain served as the response variable, while within- and between-module factors served as fixed factors, with each module and species treated as random factors. All analyses were conducted using R version 4.0.2 (R Development Core Team, 2020).

## 3 Results

### 3.1 Changes in network composition

**Figure 3:**
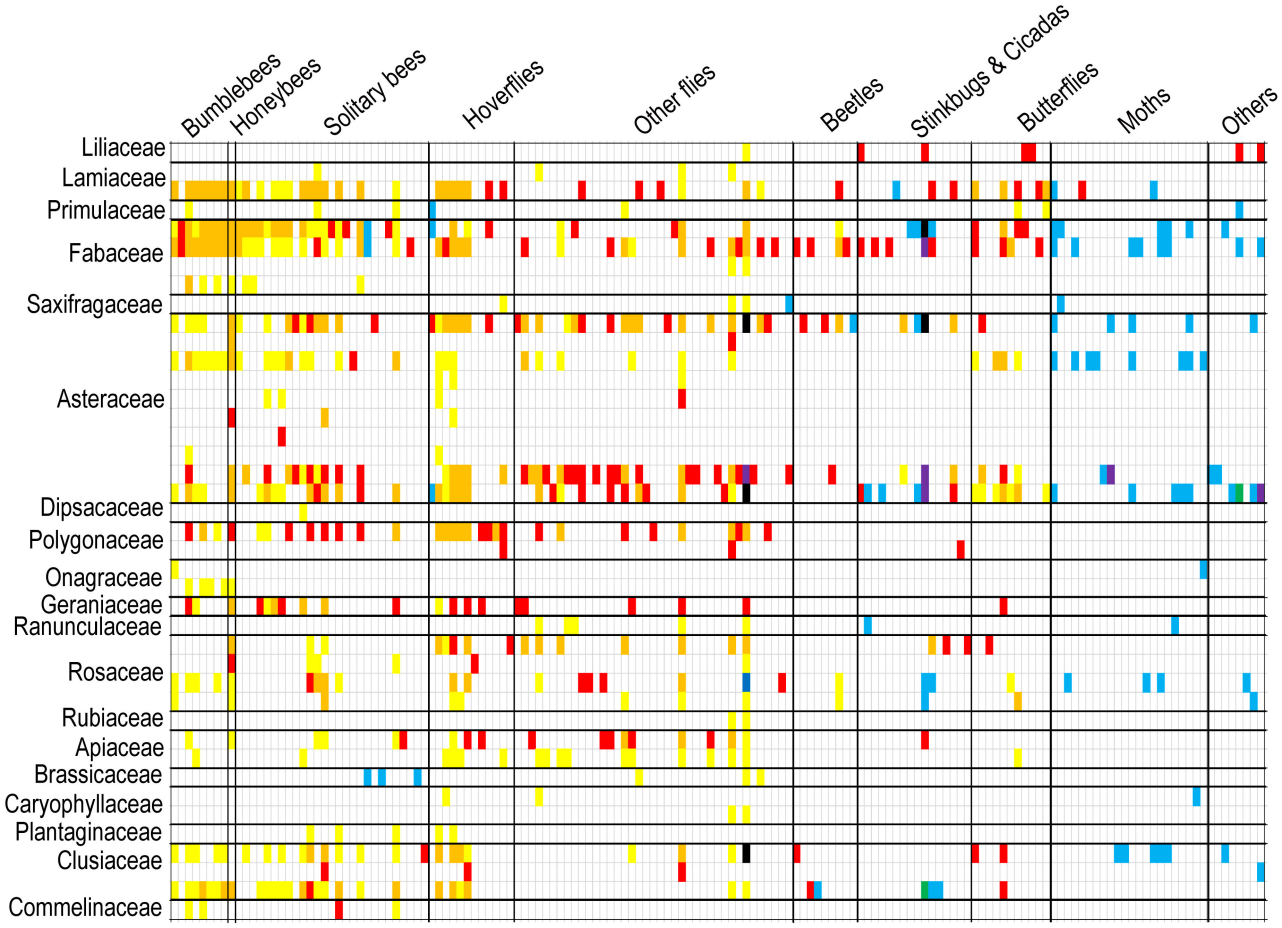
Interactions between animal and plant species of V_d_P_d_P_n_ matrix (diurnal field surveys + diurnal pollen analyses + nocturnal pollen analyses) can be depicted as matrices, where an animal species occupies a row and a plant species occupies a column, and an interaction between the two is denoted by a square. Interactions can be uncovered by different color squares (only V_d_: red squares; only P_d_: yellow squares; V_d_ overlaps P_d_: orange squares; only P_n_: blue squares; V_d_ overlaps P_n_: purple squares; P_d_ overlaps P_n_: green squares; V_d_, P_d_, and P_n_ overlap: black squares).

Diurnal field visitation (V_d_) recorded 1693 individual plant–pollinator contacts involving 115 pollinator species and 25 plant species, representing 352 specific interactions. The analysis of pollen from the body of the 272 diurnal insect specimens (P_d_, mean ± SE: 4.32 ± 0.50 per species) captured provided evidence for 413 interactions. Of these, 183 interactions were also recorded in the V_d_ dataset, while 230 were new interactions identified solely through pollen analysis. In contrast, 169 interactions were recorded exclusively during the field surveys and were not detected through pollen analysis. Analysis of pollen from the body of the 753 nocturnal insect specimens (P_n_, mean ± SE: 3.60 ± 0.21 per species) captured provided evidence for 96 interactions. Of these, 83 interactions were recorded only in nocturnal pollen (P_n_) (**Figure 2**). Therefore, the combination of field visitations and diurnal and nocturnal pollen data resulted in a 1.89-fold increase (665 interactions) (**Table 2**; **Figure 3**).

**Table 2:** Parameters describing the structure of the pollination network based on diurnal field visitation (V_d_), diurnal pollen analysis (P_d_), diurnal field visitation + diurnal pollen analyses (V_d_P_d_), nocturnal pollen analyses (P_n_), and diurnal field visitation + diurnal pollen analyses + nocturnal pollen analyses (V_d_P_d_P_n_).

	V_d_	P_d_	V_d_P_d_	P_n_	V_d_P_d_P_n_
Plant species	25	38	41	18	41
Pollinator species	115	59	115	46	153
Interactions recorded	352	413	582	96	665
Connectance	0.122	0.184	0.123	0.116	0.106
Mean plant connectivity (*S*_P_)	14.08	10.87	14.20	5.33	16.22
Mean pollinator connectivity (*S*_A_)	3.06	7.00	5.06	2.09	4.35
% Extreme pollinator specialists^#^	43.48	10.17	36.52	50.00	39.87
Nestedness based on overlap and decreasing fill (NODF)	32.69**	49.08**	42.99**	17.34 NS	36.44**
Degree centralization (DC)	0.27	0.26	0.30	0.20	0.29
Modularity (*M*)	0.33 **	0.26**	0.27**	0.48 **	0.29**
Number of significant modules	6	5	5	9	7

^#^One-link species, **P < 0.001, NS, non-significant

**Figure 4:**
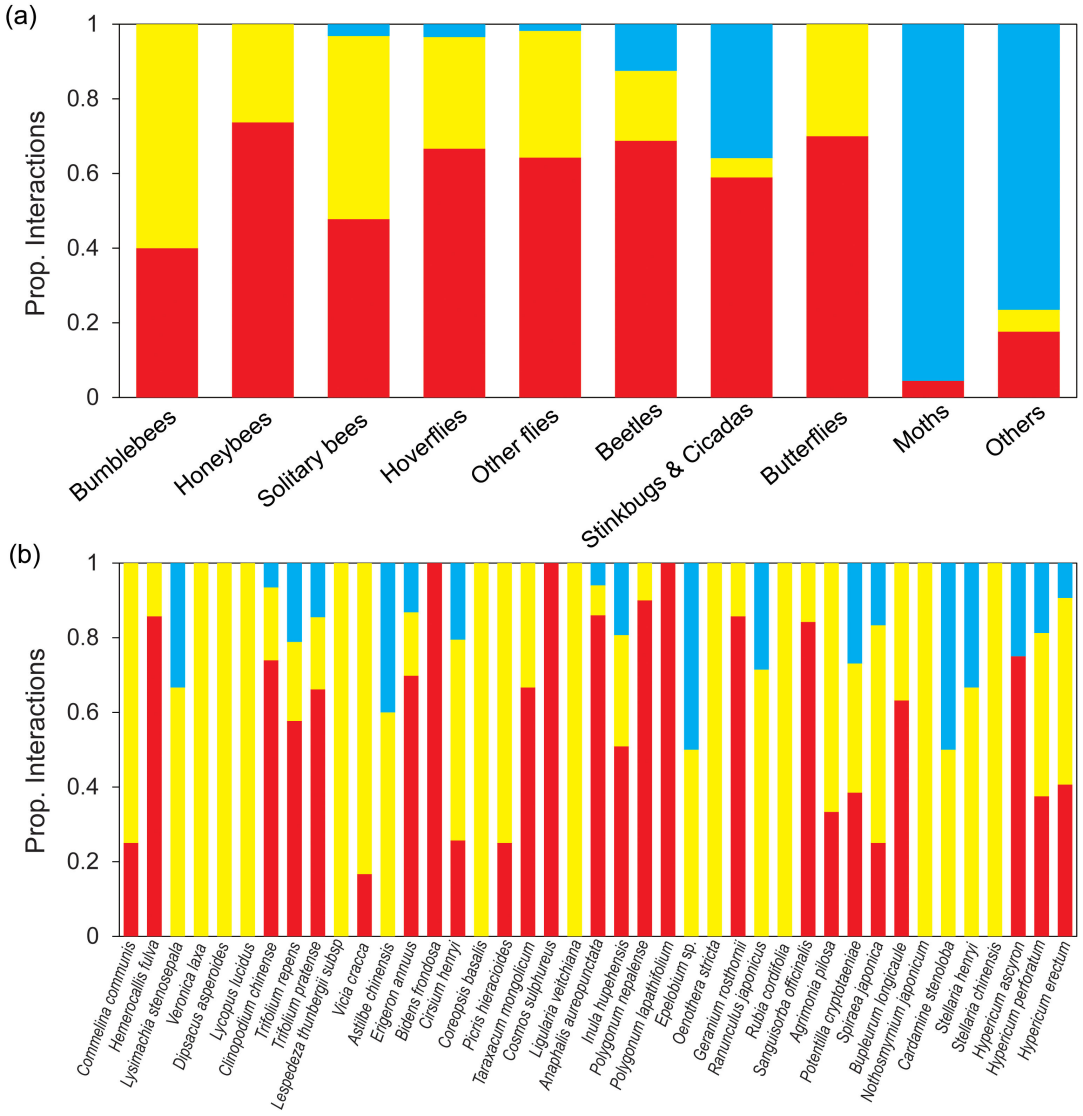
Proportion of diurnal and nocturnal interactions per pollinator groups **(A)** and plant species **(B)**. Proportion of interactions of the different pollinator groups and plant species in the diurnal visitation network (V_d_; red bars) and proportion of interactions added by diurnal pollen analysis (P_d_; yellow bars) and nocturnal pollen analysis (P_n_; blue bars).

Although diurnal field visitation as well as diurnal and nocturnal pollen analysis were sampled simultaneously and continuously throughout the active period, species tended to exhibit notably different sets of interaction partners in the visitation and pollen-load networks (**Figure 3**). However, the interaction overlap between Lamiaceae and Fabaceae plants with bumblebees and honeybees pollinator groups was relatively high in both diurnal field visitation and diurnal pollen data methods, with overlap percentages of 100% and 68%, respectively. Diurnal pollen data supplemented almost all the interactions between plant and pollinator groups, whereas nocturnal pollen data mainly supplemented interactions between plants and moths or other pollinator groups (**Figure 4A**). In terms of plants, diurnal and nocturnal pollen data supplemented the majority of interaction patterns between plant species and pollinators, except for *Bidens frondosa*, *Cosmos sulphureus*, and *Polygonum lapathifolium*. Furthermore, interactions of 39% of plant species were supplemented through diurnal and nocturnal pollen analyses (**Figure 4B**).

### 3.2 Changes in network structure

With the addition of diurnal pollen data, connectance, mean plant connectivity, and mean pollinator connectivity increased, whereas the extreme specialist pollinator species decreased by 0.8-fold (**Table 2**). After adding nocturnal pollen data, the connectance decreased 0.9-fold, mean plant connectivity went from 14.20 to 16.22, and mean pollinator connectivity went from 5.06 to 4.35, respectively. The proportion of extreme specialist pollinator species increased from 36.52% to 39.87%. The degree of centralization was low in all five matrices and increased slightly with the addition of pollen data (**Table 2**). This strengthened the differences in connectivity among species. NODF increased with the addition of diurnal pollen data but decreased with the addition of nocturnal pollen data. Except for the P_n_ matrix, the other four matrices were significantly nested (**Table 2**), likely because the nocturnal network was relatively small.

All five matrices recorded interaction rarefaction curves that exhibited a clear rise in interaction richness across the curve, with a decrease in gradation towards the end (**Supplementary Figure 1**). These rarefaction curves indicated that our sampling was relatively detailed and that we captured a significant portion of the interaction richness pool. Pollinator and plant connectivity distributions followed a truncated power law in all five matrices (**Supplementary Figure 2**). Changes in specialization and nestedness did not alter the shape of connectivity distribution.

Connectivity increased with interaction frequency (*f*), for plants and pollinators (**Supplementary Table 4**). The slopes of the three plant or pollinator *f*–*s* regression lines (V_d_, V_d_P_d_, and V_d_P_d_P_n_) were almost identical (Plant: *F_2,103_* = 0.53, *P* = 0.588; Pollinator: *F_2,379_* = 1.61, *P* = 0.202). These results have demonstrated that the relationship between *f* and *s* does not change with the addition of diurnal or nocturnal pollen data. For diurnal matrices, the pollinator increase in *s* was positively related to the number of specimens sampled (from V_d_ to V_d_P_d_: *R^2^* = 0.69, *P* < 0.0001, slope = 0.816). This suggested that connectivity could still be increased, at least for rare diurnal species. However, for nocturnal pollen analysis, we captured a large number of insects using light traps without distinguishing their pollination roles, which likely increased the capture of rare species (from V_d_P_d_ to V_d_P_d_P_n_: *R^2^* = 0.083, *P* = 0.053, slope = 0.257).

### 3.3 Module analysis of networks

The V_d_, P_d_, P_n_, V_d_P_d_, and V_d_P_d_P_n_ matrices yielded six (*M* = 0.33; *P* < 0.001), five (*M* = 0.26; *P* < 0.001), nine (*M* = 0.48; *P* < 0.001), five (*M* = 0.27; *P* < 0.001), and seven (*M* = 0.29; *P* < 0.001) significant modules, respectively. Moreover, the modularity of the network decreased with the inclusion of diurnal pollen analysis data but increased with that of nocturnal pollen analysis data (**Table 2**; **Figure 3**; **Supplementary Figure 3**).

**Figure 5:**
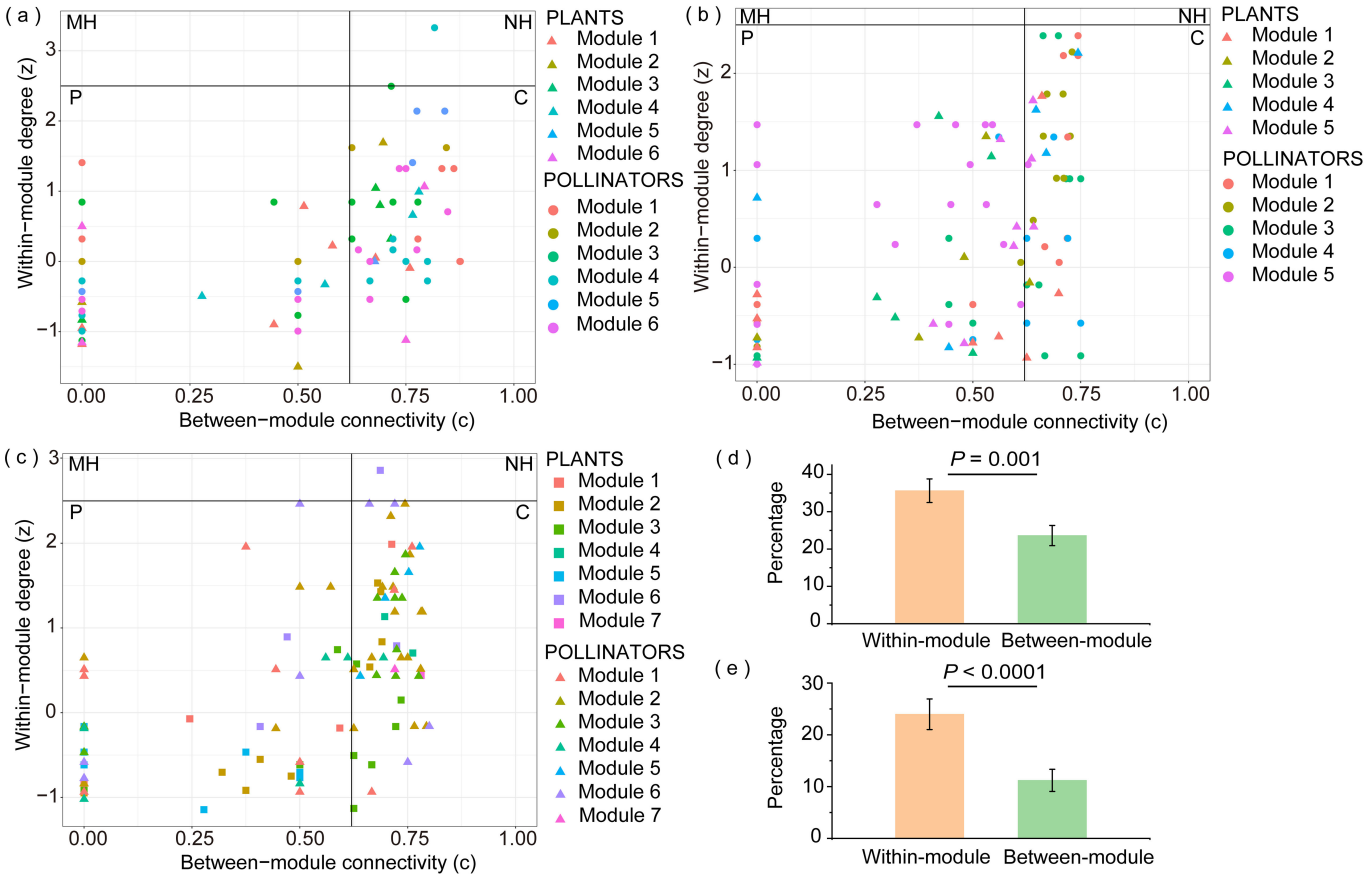
Distribution of plant and pollinator species according to their network role in the **(A)** V_d_ matrix (diurnal field visitation); **(B)** V_d_P_d_ matrix (diurnal field visitation + diurnal pollen analysis); **(C)** V_d_P_d_P_n_ matrix (diurnal field visitation + diurnal pollen analysis + nocturnal pollen analysis). MH, module hub; NH, network hub; P, peripheral; C, connector. Triangles, circles, and squares may represent more than one species. Comparison of the significant differences in the percentage of within- and between-module link gain with the addition of diurnal pollen data **(D)** and nocturnal pollen data **(E)** in species belonging to the various modules of the Dajiuhu pollination networks.

In the V_d_ matrix (**Figure 5A**), the only network hub was a pollinator species belonging to Module 4 (*Adelphocoris suturalis*). Connectors accounted for 34.8% of the total species, with 55.0% of the connector species being flies. No modules or network hubs were found in the V_d_P_d_ matrix (**Figure 5B**). Connectors accounted for 27.6% of the total, and most (69.8%) of the connector species were flies. In the V_d_P_d_P_n_ matrix (**Figure 5C**), the only network hub was a species belonging to Module 6 (*A. aureopunctata*). Connectors accounted for 34.0% of the total, and the most common connector species were bees (25.8%) and flies (31.8%). Most connector plant species exhibited high flower densities and were widely distributed across the plots. The high proportion of connector species (V_d_: 35%; V_d_P_d_: 28%; V_d_P_d_P_n_: 34%) indicated that our network was also significantly modular, but these modules were far from isolated. The additional interactions significantly occurred intramodularly (**Figures 5D, E**; **Supplementary Table 6**), indicating an increase in modularity.

Most new links shown by the diurnal pollen data (V_d_P_d_) involved species from Module 5 (**Supplementary Table 4A**; **Figure 5A**). After the addition of nocturnal pollen data and the V_d_P_d_P_n_ matrix, most new links revealed by nocturnal pollen data included species from Module 2 (**Supplementary Table 5B**; **Figure 5B**). We found that the plant species in these two modules were relatively similar. Some pollinator species overlapped, most of which were bees, butterflies, and moths with long tongues.

With the addition of diurnal pollen data, the pollination syndromes of plants and pollinators in the modules of the V_d_P_d_ matrix were better matched. Plants with higher abundance in the same meadow mostly appeared in the same module. Module 2 included seven species that were found in the same field (Meadow 2) with higher abundance, except *Coreopsis basalis*. Module 3 included seven species, four of which were found in the same field (Meadow 3) with a higher abundance. Module 4 included five plant species, with *Anaphalis aureopunctata* and *Erigeron annuus* growing together (Meadow 6) and *Hemerocallis fulva* and *Inula hupehensis* growing together (Meadow 2). With the addition of nocturnal pollen data, for the V_d_P_d_P_n_ matrix, the number of modules increased to seven, and the pollination syndromes of plants and pollinators in the modules were more closely matched. However, some species, such as *Clinopodium chinense*, *Trifolium pratense*, and *E. annuus* were separated from the original modules to form a unique module that destroyed distinctive regional components.

## 4 Discussion

The diurnal pollen data showed that new links were preferentially attached to highly connected nodes, increasing the network’s nestedness by 1.3-fold and mean pollinator connectivity from 3.1 to 5.1. This indicates that diurnal pollination added complexity and connectivity to the network without reducing the level of asymmetric specialization. These findings support the redundancy hypothesis for diurnal pollination, as the new interactions enhanced the existing network structure but did not fundamentally alter the specialization patterns. In contrast, the nocturnal pollen data revealed that nocturnal pollinators exhibited more specialized behaviors, leading to an increase in the number of extreme-specialist pollinator species. This resulted in a 0.8-fold decrease in nestedness but an increase in mean plant connectivity from 14.2 to 16.2. These findings support the complementarity hypothesis for nocturnal pollination, as the inclusion of nocturnal pollination data introduced new pollinator species and increased the overall connectivity of plants in the network.

Most plant–pollinator networks have been quantified using direct observations of contacts between visitors and flowers in the field, which is a method that has been subject to undersampling (Vázquez et al., 2009; Blüthgen, 2010; Olesen et al., 2011). The use of pollen found on insect bodies is an alternative method that may help to reconstruct a more accurate image of the entire network (Bosch et al., 2009; Gibson et al., 2011; King et al., 2013; de Manincor et al., 2020; Tourbez et al., 2023; Cirtwill et al., 2024). The pollen analysis revealed a considerable number of previously undetected interactions that were overlooked during the field visitations. The opposite was also true; a non-negligible fraction of the interactions observed in the field surveys were not detected in the pollen analyses (**Figure 3**). Cirtwill et al. (2024) also suggested that each method may favor the detection of different species and interactions and noted that pollen load observations typically reveal more interactions per individual insect than visit observations. Therefore, pollen analysis should not be regarded as a substitute for visual surveys but rather as a complementary method. Some studies thought pollen analysis was conducted to determine which flower visitors acted as potential pollinators (pollen vectors) or as cheaters (those not carrying pollen of the visited plants) (Zhao et al., 2019). However, according to the data recorded in our pollen analysis, only 5 species of insects did not carry more than 5 grains of pollen. Although some pollinators recorded visiting certain plants but did not record carrying pollen of that plant, this may be related to individual differences, and the possibility of insects carrying pollen of that plant cannot be ruled out with the increase in the number of recorded specimens. Therefore, we do not think that the role of “cheater” pollinators can be easily identified. These insects can be used as possible pollinators of plants, but it is only a matter of pollination efficiency. Nocturnal pollinators have been proven to contribute key pollination services to several wild plant families, in addition to providing functional resilience to diurnal networks (Banza et al., 2015; Knop et al., 2017; Walton et al., 2020). In this study, the pollinator species recorded in the nocturnal pollen data were almost completely different from those recorded during the day, with only eight pollinator species and 13 interactions occurring during both day and night (**Figure 3**).

A subsequent study showed that the *f*–*s* relationship and the distribution of specialization were robust to reductions of sampling effort (Nielsen and Bascompte, 2007). It remains to be seen whether increases in sampling effort lead to higher connectivity increases in rare species compared to abundant species, resulting in a flattening of the *f*–*s* relationship. In our study, the slope of the pollinator *f*–*s* regression line in the V_d_ matrix showed no significant difference in the V_d_P_d_ matrix, indicating that species with low *s* in the V_d_ matrix did not experience a greater increase in the V_d_P_d_ matrix. Another reason for our results may be that we included pollinator species that yielded no pollen records, which lowered the increase in *s* for rare species. Asymmetry in interaction networks may be explained by the distribution of species abundance, at least in part. Abundant species are highly connected because they have frequent encounters, whereas rare species are less connected because of their rarity (Vázquez et al., 2007). Vázquez et al. (2009) suggested that relative species abundance and complementarity in spatiotemporal distribution contributed substantially to the generation of observed network patterns. CaraDonna et al. (2017) proposed that species phenology and relative abundance can predict the occurrence of pairwise interactions. Overall, the species abundance was important for network construction. In our study, *s* (as obtained in V_d_P_d_) was positively correlated with both flower and pollinator abundance (**Supplementary Table 7**; pollinators: n = 115, *R^2^* = 0.79, *P* < 0.0001; plants: n = 25, *R^2^* = 0.68, *P* < 0.0001; log-transformed data), indicating the importance of abundance in the construction of plant–pollinator networks in this study area.

The addition of diurnal and nocturnal insect pollen data to diurnal visitation networks increased the number of plants and insect species and their unique interactions, which changed the composition and structure of the network. Tourbez et al. (2023) indicated that networks constructed with pollen data were more diverse in plant species and interactions, exhibiting lower modularity and specialization and higher nestedness than those of visitation networks based on field observations. Similarly, our results demonstrated that the inclusion of diurnal pollen data reduced the number of extreme specialists and modularity while increasing nestedness. This result could be attributed to diurnal pollen data capturing interactions between more abundant or generalist pollinators and plants that may not be documented in diurnal field surveys. These differences are largely explained by the greater number of interactions per individual insect revealed by pollen loads, which reflect the visits made over recent days or weeks by the insect.

The inclusion of nocturnal moth pollinators significantly alters the properties of the pollination network, leading to a decrease in total connectivity, connectance, and nestedness, while simultaneously increasing web asymmetry and modularity (García et al., 2024). The nocturnal pollen network (P_n_ matrix) contained up to 50% extreme specialists among the pollinators, and there was a 3% increase in the number of extreme specialists in the V_d_P_d_P_n_ matrix after incorporating nocturnal pollen data. The extensive specialization observed in nocturnal pollination networks has been thoroughly documented (Devoto et al., 2011; Banza et al., 2015; Borges et al., 2016), indicating that nocturnal pollinators likely developed distinct interactions with particular plant species. The reason that the connectance of the V_d_P_d_P_n_ matrix decreased after the addition of nocturnal pollen data may be that the number of total potential links increased significantly. However, the increase was relatively small in the proportion of observed links. This pattern is consistent with the notion that as networks become more specialized, the proportion of realized interactions tends to decrease, leading to lower connectance. Modularity is expected to increase with link specificity (Lewinsohn et al., 2006). In our study, the incorporation of diurnal pollen data into the V_d_P_d_ matrix resulted in a decrease in the number of modules to five. When the nocturnal pollen data were incorporated, it appears that distinct groups of pollinators began to specialize even further, forming separate modules that reflect their unique interactions with specific floral resources. This may indicate that nocturnal and diurnal pollinators exploit different niches within the broader pollination community, which can lead to increased resilience of the network as a whole while simultaneously reducing redundancy. Ultimately, the patterns of decrease and subsequent increase in modularity underscore the complex dynamics of pollination networks, where the introduction of new participants can shift network properties in unexpected ways.

Three of the modules obtained from our V_d_P_d_ matrix exhibited clear regional components. This result was consistent with most plants in these three modules growing and blooming in large numbers in a certain meadow, coupled with the high connectivity obtained in our V_d_P_d_ matrix. Modularity may be driven by the evolution among plant species in various key traits (Lewinsohn et al., 2006). We also found that most of the plants in one module matched the pollinators’ pollination syndrome. Plant species that represented mostly clustered flowers or inflorescences with less nectar production were generally clustered with medium-sized hoverflies or flies. Meanwhile, plant species that produced a certain amount of nectar and had corolla tubes were clustered with bees and butterflies with long tongues. There is a clear seasonal component within the module and that pollinator distribution is mainly driven by flowering phenology (Bosch et al., 1997, Bosch et al., 2009; Ramosamp, 2018). However, we only selected peak flowering with the highest plant abundance, and most of the plants had overlapping floral phenologies. Therefore, seasonality had little effect. We found that the regional component was no longer evident after the addition of nocturnal pollen data. This may be because of the high flight ability of nocturnal moths. Pollen is carried over greater distances by moths than by most diurnal insect pollinators (Devoto et al., 2011; Macgregor et al., 2017).

Unfortunately, nocturnal field visitations had to be overlooked because of the intrinsic difficulty of field experimentation at night, which may have had some impact on our network. It is considered that the number of pollen carried by insects to different plants may not only be related to the number of visits, but also to the pollen yield of different plants and the pollen-carrying ability of pollinators. Only qualitative binary matrixes were constructed to analyze the pollination networks obtained by superimposing data from different methods. Constructing a better quantitative pollination network may need to consider information such as the number of pollen grains deposited on the stigma after a single visit by the pollinator. Simultaneously, since this study was conducted in a specific habitat, our results have certain limitations and cannot be easily generalized to other habitats or broader ecosystems. This study provides a preliminary exploration of the impact of incorporating both diurnal and nocturnal pollen analysis into daytime flower visitor observations on pollination networks. However, research conducted in different locations and habitats may yield different results. Therefore, we suggest that future studies be carried out in various habitats and across a broader geographical range to further extend our findings. We also recommend the use of more data points and longer monitoring periods to attain more comprehensive and representative results.

In addition, we would like to emphasize that this study focuses on the role of sampling completeness in network structure by constructing a multi-layered qualitative binary matrix of plant-pollinator interactions. Although we utilized the same visitation data as Teng et al. (2024), our research objectives and methodologies exhibit significant differences. While Teng et al. (2024) concentrated on quantitatively comparing the diurnal and nocturnal pollination networks to reveal differences in composition and structure, as well as to explore their respective contributions to plant reproduction, our study demonstrates the profound impacts of sampling methods and detailed recording on pollination network structure. By progressively integrating different data sets, we provide a new perspective on how sampling completeness can influence pollinator monitoring schemes. Thus, our findings not only contribute to a more comprehensive understanding of this topic but also have practical implications for enhancing conservation efforts and future ecological research.

## 5 Conclusion

Our study added both diurnal and nocturnal insect pollen data to visitation data and constructed a more informative plant–pollinator network. Transect and insect pollen data have different utility and efficacy for monitoring different aspects of plant–pollinator interaction biodiversity. Based on the species composition of the pollen load, we unveiled a significant number of interactions undetected in diurnal plant-centered field visitations that resulted in significant changes in some fundamental properties of the network structure. The insect pollen data revealed interactions involving rare plant species and a greater diversity of new connections between high-abundance plants and pollinators. Simultaneously, owing to the large differences between nocturnal and diurnal animal taxa, the addition of nocturnal insect pollen data increased the number of pollinator species. By including nocturnal pollination data, we were able to uncover significant interactions and structural changes that would have remained hidden if we had only focused on diurnal pollination. This approach reveals the full complexity of the pollination network, demonstrating how nocturnal pollinators contribute uniquely to the ecosystem. Specifically, the inclusion of nocturnal data allowed us to observe an increase in the number of extreme-specialist pollinator species, which significantly altered the network’s nestedness and connectivity. Our study introduces a novel approach by integrating both diurnal and nocturnal pollen data to analyze pollination networks. Understanding the complete interaction network of pollinators in the community will help us explore the true niche partitioning in plants and pollinators and their evolutionary trajectories. In turn, this information will provide more detailed and feasible strategies for facing a series of threats from environmental changes. In the future, we hope to increase awareness of the importance of network integrity when revealing certain rules of pollination.

## Data Availability

The original contributions presented in the study are included in the article/**Supplementary Material**, further inquiries can be directed to the corresponding author/s.

## Supplementary material

Supplementary Figure 1 Interaction-based rarefaction curves (mean ± 95% confidence intervals) showing the expected interaction richness for diurnal field visitation (V_d_), diurnal pollen analyses (P_d_), diurnal field visitation + diurnal pollen analyses (V_d_P_d_), nocturnal pollen analyses (P_n_) and diurnal field visitation + diurnal pollen analyses +nocturnal pollen analyses (V_d_P_d_P_n_) data. The number of interactions (x-axis) observed is represented by the solid portion of each colored line, whereas the dashed portion indicates extrapolation in the rarefaction analysis using the R package iNEXT. 95% confidence intervals are shown as shaded areas.

Supplementary Figure 2 Cumulative distribution of connectivity (number of links per species) for pollinators and plants in Dajiuhu, based on diurnal field visitation (V_d_), diurnal pollen analysis (P_d_), diurnal field visitation + diurnal pollen analysis (V_d_P_d_), nocturnal pollen analysis (P_n_), and diurnal field visitation + diurnal pollen analysis + nocturnal pollen analysis (V_d_P_d_P_n_). Each circle may represent more than one species. Plant connectivity distribution follows a truncated power law (V_d_ matrix: γ = 1.42; P_d_ matrix: γ = 1.44; V_d_P_d_ matrix: γ = 1.41; P_n_ matrix: γ = 1.57; V_d_P_d_P_n_ matrix: γ = 1.39), and pollinator connectivity also distribution a truncated power law (V_d_ matrix: γ = 1.82; P_d_ matrix: γ = 1.47; V_d_P_d_ matrix: γ = 1.64; P_n_ matrix: γ = 2.04; V_d_P_d_P_n_ matrix: γ = 1.70).

Supplementary Figure 3 Comparison of observed with null model Patefield in all five matrices. Non-overlapping with the density curve suggests a significant difference between the network parameters of the observed network and those of the random network.

Supplementary Figure 4 Interactions between animal and plant species can be depicted as matrices, where an animal species occupies a row and a plant species occupies a column, and an interaction between the two is denoted by a square. (a) V_d_P_d_ matrix (diurnal field surveys + diurnal pollen analyses) and (b) V_d_P_d_P_n_ matrix (diurnal field surveys + diurnal pollen analyses + nocturnal pollen analyses). The structure of matrices follows the modular pattern, and the interactions of the same module are put in one black box. Interactions can be uncovered by different color squares (only V_d_: red squares; only P_d_: yellow squares; V_d_ overlaps P_d_: orange squares; only P_n_: blue squares; V_d_ overlaps P_n_: purple squares; P_d_ overlaps P_n_: green squares; V_d_, P_d_, and P_n_ overlap: black squares).

Supplementary Table 1 For the plant species found in the three datasets, corresponding module numbers have been indicated **Supplementary Figure S3**.

Supplementary Table 2 List of all recognizable taxonomic units, corresponding family and order, identified in the diurnal and nocturnal dataset. The module numbers have been indicated.

Supplementary Table 3 The data of the five plant–pollinator qualitative binary matrices: diurnal field visitation (matrix V_d_), diurnal pollen analysis (matrix Pd), diurnal visitation and pollen analysis (matrix V_d_P_d_), nocturnal pollen analysis (matrix P_n_), and all three types of data (matrix V_d_P_d_P_n_).

Supplementary Table 4 Linear regression was used to explore the relationship between *f* (interaction frequency and number of observed flower visits) and *s* (connectivity) for both plants and pollinators.

Supplementary Table 5 Within- and between-module link gain with the addition of diurnal pollen data (a) and nocturnal pollen data (b) in species belonging to the various modules of the Dajiuhu pollination networks.

Supplementary Table 6 Comparison of the significant differences in the percentage of within- and between-module link gain with the addition of diurnal pollen data and nocturnal pollen data in species belonging to the various modules of the Dajiuhu pollination networks. GLMM: generalized linear mixed models. Family: binomial (logit). Test statistics (test stat.): z value for glmer.

Supplementary Table 7 Linear regression was used to explore the relationship between the species abundance and *s* (connectivity, obtained in V_d_P_d_) for both plants and pollinators. The floral abundance of plant species here refers to the floral visual units in each sample plot (**Supplementary Table 1**), and the abundance of pollinators refers to the number of recorded individuals of the pollinators. All censuses were log-transformed prior to analysis.
