# Supplementary figures and images for: Constructing more comprehensive pollination networks: integrating diurnal and nocturnal pollen data with visitation in a subalpine wetland community
Source: Front Plant Sci. 2024 Oct 8;15:1464970. doi: 10.3389/fpls.2024.1464970 (PMC11494514; doi:10.3389/fpls.2024.1464970)

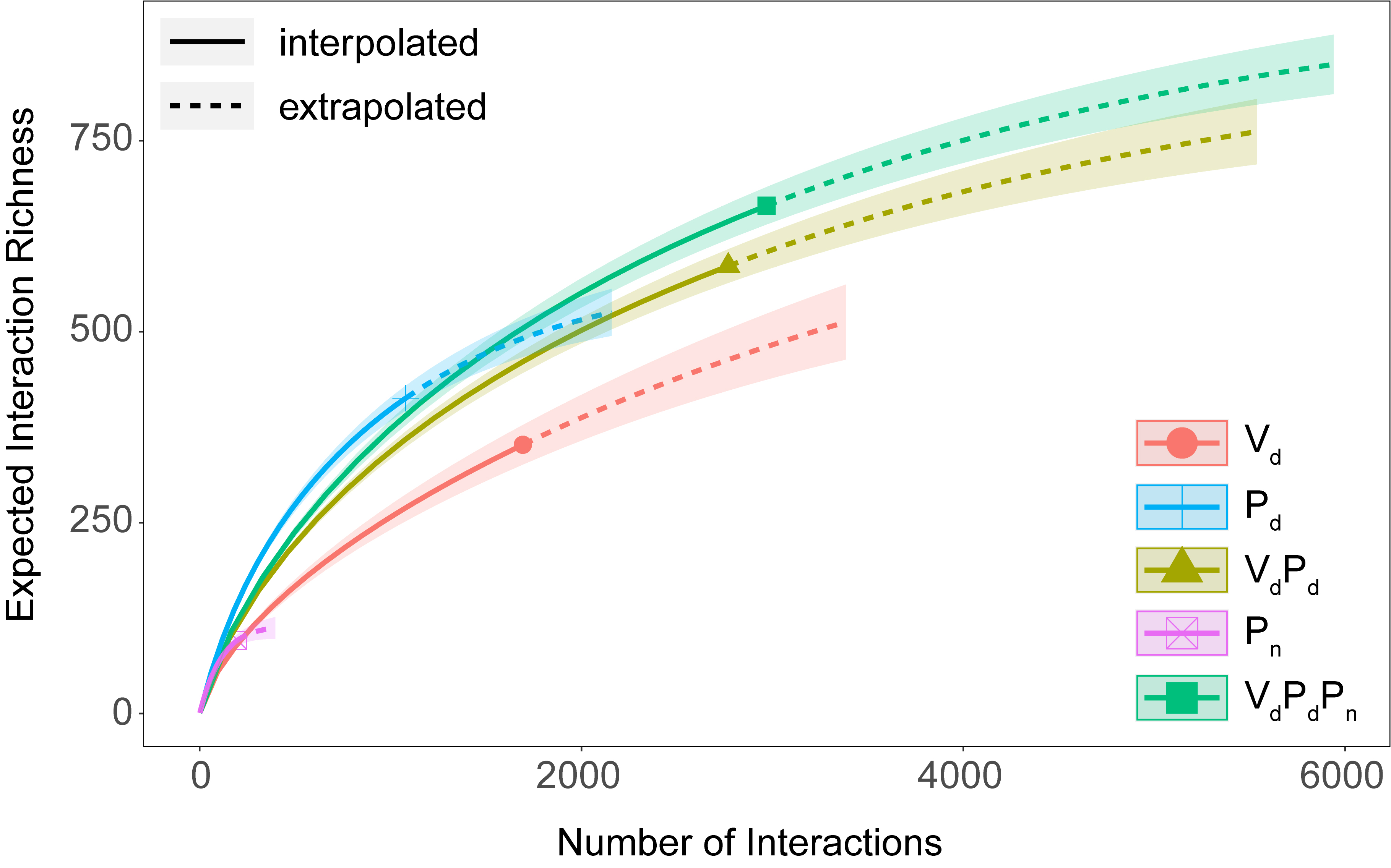

Supplement: Supplementary Figure 1 — Interaction-based rarefaction curves (mean ± 95% confidence intervals) showing the expected interaction richness for diurnal field visitation (Vd), diurnal pollen analyses (Pd), diurnal field visitation + diurnal pollen analyses (VdPd), nocturnal pollen analyses (Pn) and diurnal field visitation + diurnal pollen analyses +nocturnal pollen analyses (VdPdPn) data. The number of interactions (x-axis) observed is represented by the solid portion of each colored line, whereas the dashed portion indicates extrapolation in the rarefaction analysis using the R package iNEXT. 95% confidence intervals are shown as shaded areas. [file Image1.jpeg]

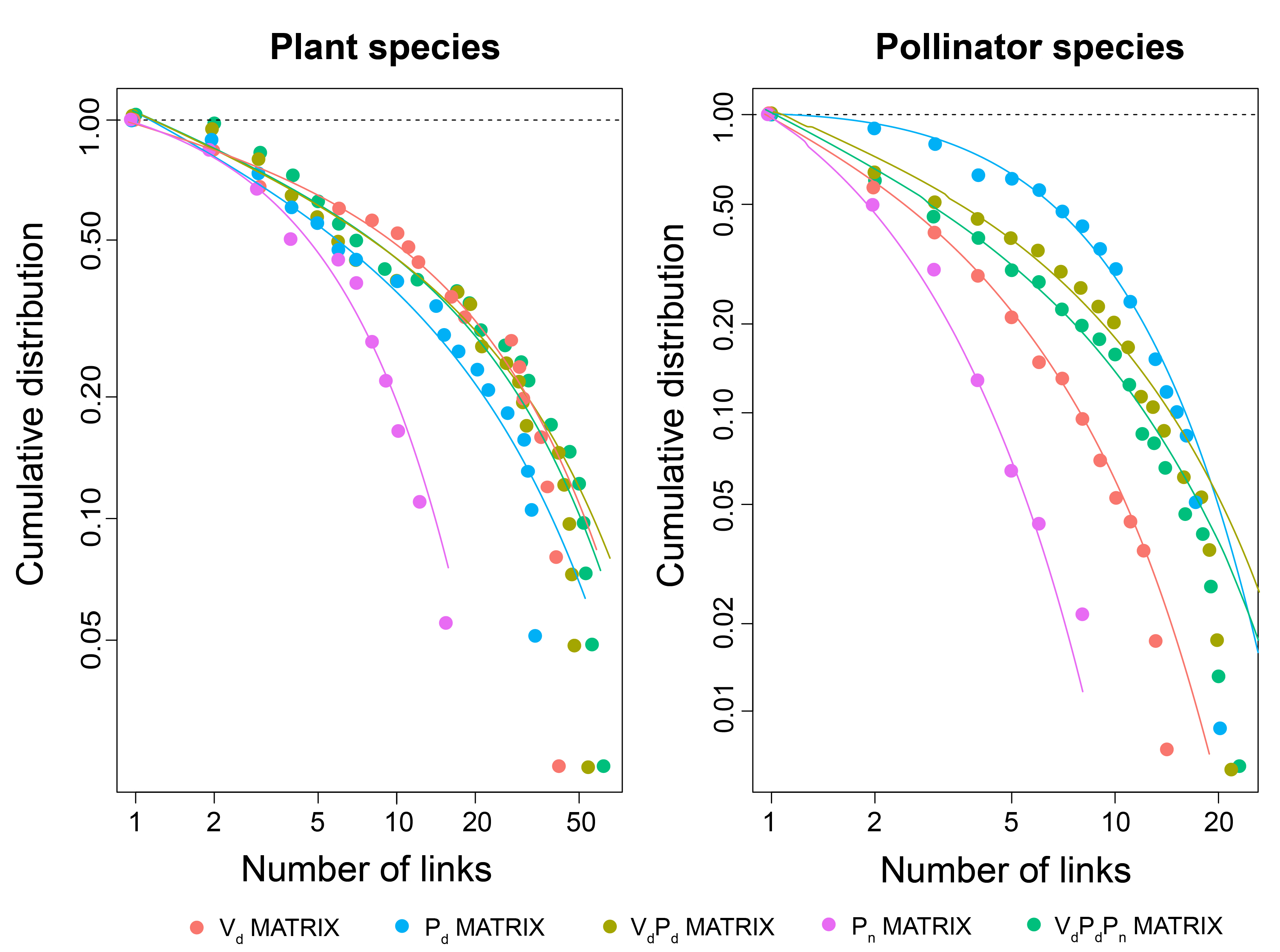

Supplement: Supplementary Figure 2 — Cumulative distribution of connectivity (number of links per species) for pollinators and plants in Dajiuhu, based on diurnal field visitation (Vd), diurnal pollen analysis (Pd), diurnal field visitation + diurnal pollen analysis (VdPd), nocturnal pollen analysis (Pn), and diurnal field visitation + diurnal pollen analysis + nocturnal pollen analysis (VdPdPn). Each circle may represent more than one species. Plant connectivity distribution follows a truncated power law (Vd matrix: γ = 1.42; Pd matrix: γ = 1.44; VdPd matrix: γ = 1.41; Pn matrix: γ = 1.57; VdPdPn matrix: γ = 1.39), and pollinator connectivity also distribution a truncated power law (Vd matrix: γ = 1.82; Pd matrix: γ = 1.47; VdPd matrix: γ = 1.64; Pn matrix: γ = 2.04; VdPdPn matrix: γ = 1.70). [file Image2.jpeg]

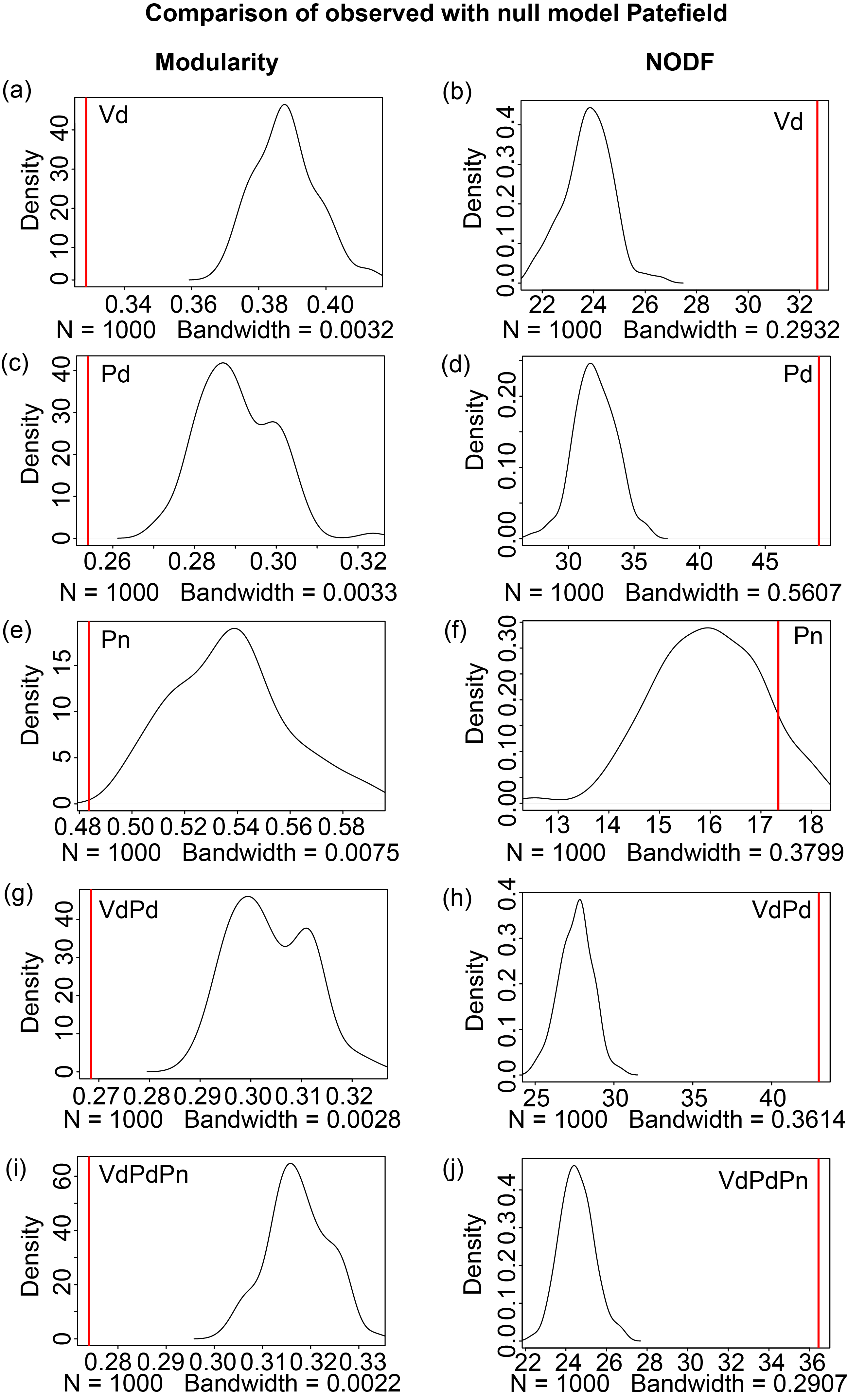

Supplement: Supplementary Figure 3 — Comparison of observed with null model Patefield in all five matrices. Non-overlapping with the density curve suggests a significant difference between the network parameters of the observed network and those of the random network. [file Image3.jpeg]

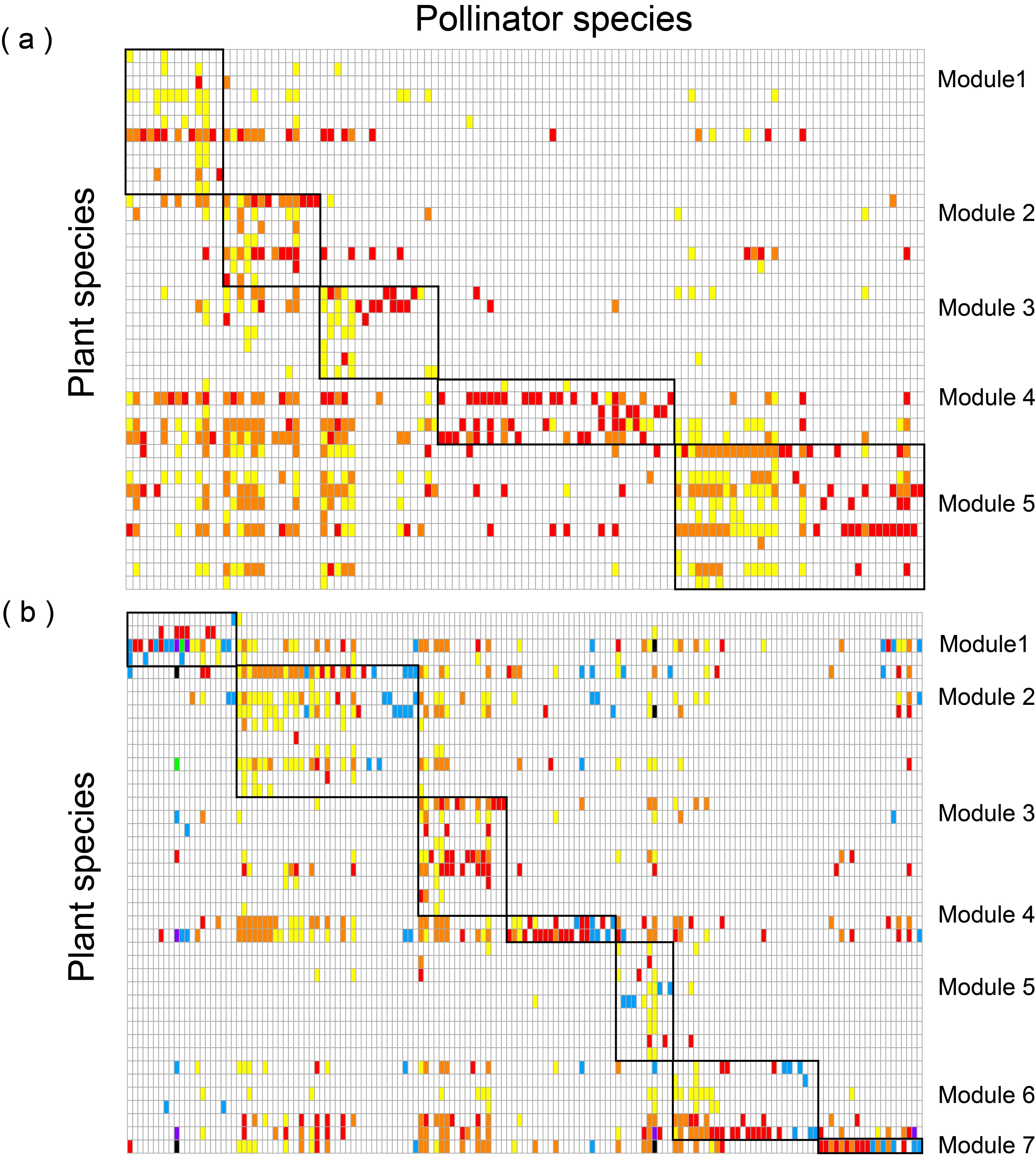

Supplement: Supplementary Figure 4 — Interactions between animal and plant species can be depicted as matrices, where an animal species occupies a row and a plant species occupies a column, and an interaction between the two is denoted by a square. (a) VdPd matrix (diurnal field surveys + diurnal pollen analyses) and (b) VdPdPn matrix (diurnal field surveys + diurnal pollen analyses + nocturnal pollen analyses). The structure of matrices follows the modular pattern, and the interactions of the same module are put in one black box. Interactions can be uncovered by different color squares (only Vd: red squares; only Pd: yellow squares; Vd overlaps Pd: orange squares; only Pn: blue squares; Vd overlaps Pn: purple squares; Pd overlaps Pn: green squares; Vd, Pd, and Pn overlap: black squares). [file Image4.jpeg]
