# Supplementary material for: Constructing more comprehensive pollination networks: integrating diurnal and nocturnal pollen data with visitation in a subalpine wetland community
Source: Front Plant Sci. 2024 Oct 8;15:1464970. doi: 10.3389/fpls.2024.1464970 (PMC11494514; doi:10.3389/fpls.2024.1464970)
Supplement: Supplementary file 6 [file Table2.docx]

**Supplementary Materials**

**Diurnal field visitation**

Each census was conducted over a span of 3–4 consecutive days, with a gap of 3–5 days before commencing the next census. To ensure systematic sampling, each meadow was divided into four quadrants. During each 15-min observation period, we randomly selected one quadrant within the meadow as the focal quadrant. The remaining quadrants were then sampled in a random order. This process was repeated for all six meadows. To eliminate bias, we alternated the starting meadow on successive observation days, following a consistent pattern (CaraDonna et al., 2017). During each observation period, we walked at a consistent speed around the focal quadrant, meticulously documenting all observed plant-pollinator interactions. For every visitor collected, we recorded the corresponding plant species. All observations were conducted between 0900 and 1600 h, specifically during weather conditions that were favorable for pollinator activity, characterized by low wind speeds and the absence of snow or rain.

**Plant abundance**

We considered a floral visual unit, which acted as a functional unit for pollinator attraction, to be a solitary flower (e.g., *Geranium rosthornii*) or an inflorescence (e.g., *Inula hupehensis*) with individual florets clustered together and which cannot be easily counted in the field. The average abundance of each plant in the sample was calculated (excluding plant species for which no visitors were recorded). Each plant species was collected in triplicate and preserved as specimens in the Herbarium of Wuhan University.

**Pollen analysis**

To facilitate pollen analysis, a pollen reference collection was prepared by sampling pollen from each flower species present at the sites, fixing them on microscope slides, and taking photos under a microscope (Binocular Nikon E100 microscope, Tokyo, Japan) at ×400 magnification. To sample the pollen carried by flower visitors, insects were softened in a relaxing chamber for 24 h before handling (Devoto et al. 2011). We added 70% ethanol to submerged insect bodies in tubes (Hymenoptera were washed without corbicula and Lepidoptera were washed without wings). Pollen grains attached to the bodies of these insects were detached using an ultrasonic cleaner for 4 min (40 kHz; JP-010T, Skymen Cleaning Equipment Shenzhen Co., Ltd., Shenzhen, China) (Gong et al., 2016). We washed each insect 1–10 times to ensure all pollen grains were removed. The pollen suspension was then placed into 2 mL tubes, which were then centrifuged for 2 min, until all the pollen was deposited on the bottom. The supernatant was discarded and Alexander’s solution was added to the centrifuge tube. The solution was mixed with a vortex oscillator for 30 s, removed with a pipette, dropped on a slide, and then examined under a microscope for identification and counting of pollen grains. We established the presence of five pollen grains from a given plant species in our pollen counts as proof of visitation to that species (Knop et al., 2017). Although we did not record the timing of pollen deposited on the insect body, we assumed that the diurnal and nocturnal activity of those insects meant that they were likely to spread pollen to plants in both periods.

**References**

Devoto, M., Bailey, S., Memmott, J., 2011. The ‘night shift’: nocturnal pollen-transport networks in a boreal pine forest. Ecological Entomology 36, 25–35. https://doi.org/10.1111/j.1365-2311.2010.01247.x.

CaraDonna, P. J., Petry, W. K., Brennan, R. M., Cunningham, J. L., Bronstein, J. L., Waser, N. M., et al. (2017). Interaction rewiring and the rapid turnover of plant–pollinator networks. *Ecol Lett* 20, 385–394. doi: 10.1111/ele.12740

Gong, Y.-B., Yang, M., Vamosi, J. C., Yang, H.-M., Mu, W.-X., Li, J.-K., et al. (2016). Wind or insect pollination? Ambophily in a subtropical gymnosperm *Gnetum parvifolium* (Gnetales): Ambophily in *Gnetum*. *Plant Species Biol.* 31, 272–279. doi: 10.1111/1442-1984.12112

Knop, E., Zoller, L., Ryser, R., Gerpe, C., Hörler, M., Fontaine, C., 2017. Artificial light at night as a new threat to pollination. Nature 548, 206–209. https://doi.org/10.1038/nature23288
